# Editorial: Next Generation Agriculture: Understanding Plant Life for Food, Health and Energy

**DOI:** 10.3389/fpls.2020.01238

**Published:** 2020-08-12

**Authors:** Domenico De Martinis, Edward P. Rybicki, Nicola Colonna, Eugenio Benvenuto, Briardo Llorente

**Affiliations:** ^1^ ENEA Italian National Agency for New Technologies, Energy and Sustainable Economic Development, Rome, Italy; ^2^ Biopharming Research Unit, Department of Molecular and Cell Biology, University of Cape Town, Cape Town, South Africa; ^3^ ARC Center of Excellence in Synthetic Biology, Macquarie University, Sydney, NSW, Australia; ^4^ CSIRO Synthetic Biology Future Science Platform, Sydney, NSW, Australia

**Keywords:** climate change, agriculture, bioproducts, food, biofuel, bioeconomy, artificial intelligence, molecular farming

Current global population growth and the associated increasing demands on farming, together with the threat of climate change and the need for better environmental protection, pose formidable challenges for the agriculture of the future. Crop productivity is already reaching high capacity but will have to increase perhaps by as much as 100% to sustain a world population of nearly 10 billion people by 2050. These global challenges are also not evenly distributed. While developed countries face the needs derived from an aging population, highly urbanized areas, and stagnation of cultivated land, developing nations are blooming in terms of population growth, the building of infrastructure, and the use of land for agriculture. Less developed countries, on the other hand, also face demographic growth but generally lack modern infrastructure and efficient farming practices required for the expansion of agricultural production. Climate change also afflicts different regions of the world with varying intensity and in very diverse ways, generating complex effects on agricultural systems, which remain hitherto unpredictable. This Research Topic provides a perspective of how and where agriculture will be conducted in the future, what will be cultivated, and for what purpose.

Studies published in this topic provide an overview of how different technologies and research areas may converge in agricultural innovation. Biotechnology could improve “intrinsically” crop performance (Fulgosi and Vojta), make plant crops more climate-resilient (Soto et al.; Gonzalo et al.), productive (Mayta et al.; Grossi et al.), and nutritious (Pannico et al.; Yuan and Li; Ferrero et al.), as well as less dependent on the use of agrochemicals (Fabian et al.), and tailored for biofuel production (Gangwar and Shankar).

Approaches dedicated to highly technologically intensive disciplines could be of great support to agriculture. Artificial Intelligence could play a game-changing role in the automation of agricultural practices (Jin et al.; Fabris et al.), freeing farmers from the workload of traditional agriculture and from the variability that still shapes the output of agricultural production.

Future agriculture is expected to produce food, energy, pharmaceuticals, and other high-value commodities, and may take place beyond traditional cultivated lands. In the past, humanity learned to claim agricultural land by draining and terracing. Now, climate change and scarcity of arable land might lead future agriculture to respond to production needs (Pourkheirandish et al.) by moving to more extreme environments (Pappalardo et al.), taking advantage of algae cultivation (Molino et al.), and merging technologies to create greenhouses to enable sustainable agriculture in desert areas (Lefers et al.). Biocontainment approaches to enable molecular farming in large-scale field conditions are discussed in this issue (Clark and Maselko). Concurrently, we have to consider that, in the near future, more crops would be grown also in urban environments, with little or no ground available. Urban farming in cities might help reduce the environmental impact of agriculture while contributing to sustainably achieving food security.

Agriculture with no-land is already happening and will eventually evolve into agricultural systems to support human on earth and beyond, on the International Space Station^1^, and for space exploration^2^.

This editorial work has been implemented in the timeframe Fall 2019–Spring 2020 on the rampage of the COVID-19 pandemic. Counting the accepted papers only, this Research Topic involved 47 laboratories, 116 authors, and 39 referees from 24 countries worldwide (Argentina, Brazil, Mexico, Cuba, USA, Australia, Japan, South Korea, Taiwan, China, India, Israel, Saudi Arabia, South Africa, Turkey, Cyprus, Croatia, Italy, Spain, Portugal, Czech Republic, Germany, UK, Poland). Working on future knowledge during a global emergency situation with so many people in lockdown has not been easy, both mentally and practically. Now that the emergency sanitary phase seems to be controlled, a lesson about our ability to continuously provide society with basic needs, such as energy, water, and food, has been clearly highlighted. The issue of food security that has been largely neglected in recent years in developed countries has returned to the fore and governments’ agenda. The primary sector must continue to ensure healthy and sufficient food for all, even during crises like the one we have experienced. When the movement of people and goods is stopped, it is of paramount importance to have local food production capacity to cope with the unavailability of external sources. The agriculture of the future will have to sustain a world population with different needs and opportunities and provide resources also in case of a global crisis while at the same time reducing environmental impact in the general upheaval of climatic conditions.

To achieve this, advances at the frontiers of plant science will become essential. Knowledge efforts in the field of physical–chemical life sciences applied to the agricultural production sector must be supported to increase the resilience of the global agri-food system. These include expanding the use of plants for the production of novel materials, complex chemicals, pharmaceuticals and biologics, and bioenergy, as well as understanding how to improve our farming practices toward a circular economy. Next-generation agriculture will certainly shape our future. It will take advantage of Smart and Molecular Farming, Data, and Artificial Intelligence; it will move into the cities and go vertical, occur in extreme environments, and support the human conquest of extraterrestrial space.

## Author Contributions

The authors contributed equally to the work.

## Conflict of Interest

The authors declare that the research was conducted in the absence of any commercial or financial relationships that could be construed as a potential conflict of interest.

## Footnotes

1 Meals Ready to Eat: Expedition 44 Crew Members Sample Leafy Greens Grown on Space Station NASA Newsletters https://www.nasa.gov/mission_pages/station/research/news/meals_ready_to_eat

2 Ground Demonstration of Plant Cultivation Technologies for Safe Food Production in Space https://eden-iss.net/

